# Dental Hygiene

**Published:** 1871-02

**Authors:** H. L. Ambler


### DENTAL HYGIENE.

BY H. L. AMBLER, D. D. S., M. D.

Inasmuch as the present luxurious habits of man, and his artificial modes of living elevate him to the highest summit of perfection, they also lead him to the greatest physical and moral vicissitudes, and not infrequently tend to injure, and even destroy the health and life of the teeth; which are considered as an example of nature's perfect and most precious works of utility and beauty. In common with the entire constitution of man, the teeth are obliged, notwithstanding the remarkable health and strength with which they have been endowed by nature, to yield to many causes which as frequently injure and destroy them, therefore we can not too strongly reprehend the bad habit of those who after meals torment their teeth and gums, by picking with a knife, a pair of scissors, pins, needles, toothpicks of ivory, bone, silver, or gold. The best toothpicks are soft wood, or quills not boiled, as then they are not deprived of that oily substance which gives them softness and elasticity. The toothpick should be ready and be used thoroughly after eating, any time of day or night. One of the great secrets for the preservation of the teeth, is avoiding everything likely to injure them, and therefore we must observe the following: never bite or crack hard bodies with them; never make a corkscrew or vise of the jaws; never bite thread or similar substances; never allow food to remain in cavities or between the teeth; never use such preparations as soot, charcoal, coral, coarse pumice stone, acid elixirs, and tinctures of any kind; never take ice-cold water or food immediately after partaking of hot food or drink; never reside in low humid places, or in localities subject to sudden and great variations of temperature; never make too free use of mineral waters, as they are apt to set the teeth on edge and cover them with a blackish coat; never eat an excess of confectionery, and shun such employment as requires the frequent handling of mercury or other metallic substances, which, on being reduced to vapor, alter the teeth in a remarkable and serious manner.

It is a subject of regret that persons wise and cautious on most other subjects, should manifest such indifference in selecting the means of preserving the teeth from premature disease, pain, and death. The management of the teeth should not be entirely limited to the dental surgeon; it will be useful to every human being to know how to take a rational and daily care of these important organs. Dr. Brown says:

"Be wise in time, 'tis folly to delay;
Cast all your vile cosmetic drugs away;
Exchange the shallow artifice of dress,
For nature's more enchanting loveliness,
And know that blooming health alone abides,
Where chaste and temperate cleanliness resides."

It is a fact known from observation, that those who have paid much attention to their teeth, and from early youth have attended to cleansing them frequently, regularly, and thoroughly, have altogether far better ones than those who neglected this important practice, or have attended to it at irregular and uncertain times. Nor could it be otherwise, for upon the presence of putrid foreign matter, vitiated mucus and saliva, do many of the diseases of these organs depend. The fact then, that cleanliness of the teeth is essential to their health and preservation being established, a motive sufficiently strong for its observance is no longer wanting. To a pleasing and agreeable expression of the countenance, a clean and healthy denture is of the greatest consequence, even if it lacks that perfect regularity which is desirable, if it only be free from all extraneous matter, its appearance will never be disgusting. However particular persons of either sex may be about dress, though they be decked with gold and sparkle with diamonds, if unmindful of their teeth, they fail to excite those feelings of admiration which they otherwise might. It is not only essential to personal appearance but to comfort, and this ought to be a sufficient inducement to the regular observance of such means as are necessary to keep the mouth and teeth well cleansed. The frequent and thorough cleansing of these organs is of the greatest importance to insure a pure breath, and those who neglect such measures must necessarily breathe contamination, the exhalations of which are quite perceptible to those around. In proportion as the teeth are unclean, especially in those of delicate constitutions, is the appetite impaired and the digestive organs weakened; and thus the capability of enjoying life is often greatly diminished.

Among the toilet articles which may injure the teeth are the several kinds of paints, waters, and pomades intended to remove freckles from the face, or dye the hair. Most of these cosmetics contain astringent and even caustic properties, such as antimony, bismuth, the hydrochlorates of lead and mercury—substances which act directly upon the teeth, to which they are carried by the lymphatic vessels which ramify upon the mucous membrane enveloping the necks of the teeth, and entering the alveolar cavities. Those who from position feel compelled to use beautifiers for head or face, should confine themselves to such as are purely vegetable, for these are less injurious to the teeth.

The habit of applying dentifrices to the teeth with a cloth, or the corner of a towel, is not a good one, for the pressure only tends to force the accumulations between the teeth, rendering it more difficult to dislodge them. Experience is in favor of the brush, which can be directed against the most distant ones, and penetrate the intervals which separate them, brushing them thoroughly both outside and inside, parallel and transverse with their axis.

The constant use of soap for cleansing these organs is somewhat objectionable, for it will eventually give them a yellowish shade, which is one of the things to be prevented. Table salt can not be used with perfect safety, for it acts much like other powders, and besides it is composed of soda and hydrochloric acid, whose action can not be nugatory in whitening the teeth. Its employment also occasions abundant salivation, which, useful enough in some circumstances, is foreign to the end we now have in view.

In subjecting the mouth early to the rules of cleanliness, and to the different hygienic precautions which we have described, we most assuredly place the teeth in the condition most favorable to their preservation; but to obtain the full efficacy, these precautions should be regular and continued. Thus may a great number of the diseases of the teeth be nearly or quite prevented. Then we say never neglect those cares of neatness which form the daily toilette of the mouth, for they are simple and easy to be performed, when once accustomed to them. A beautiful denture constitutes a charm which is hardly excelled by a noble and well cultivated mind. The preservation of these organs should be an object of solicitude with every one, feeling and knowing that one natural tooth is of more value than a full set of artificial ones. One thing much to be regretted both by operator and patient, is that people are in the habit of giving the teeth hardly a thought until they begin to ache.

No one doubts the importance of a true knowledge of the mental phenomena and of the laws by which they are regulated; neither should they doubt the importance of the care of the human teeth, of which we have the unlimited and sovereign control, inviting to practical application and meditation of the highest moment, in favor of economy, health, beauty, strength, and longevity, besides being an auxiliary to every art, science, and accomplishment in society. If, then, health can be improved and disease averted by means so easy and simple; if the growth of the body and the enjoyment of life can be so materially promoted; if the contour of the countenance, the improvement of the voice, and a more complete enunciation can also be effected by such means, who would not adopt this plan, thereby establishing in their highest perfection, a mind unfettered by a constant source of irritation, and a body impervious to those tortures which, with all the vigor of electric shocks, are darted through the medium of the nervous system, from a disordered mouth to the most remote part of the body; distressing in infancy, miserable in youth, and mortifying the remainder of life, in spite of all that can be done.

